# Supplementary material for: Predictors and incidence of depression and anxiety in women undergoing infertility treatment: A cross-sectional study
Source: PLoS One. 2023 Apr 13;18(4):e0284414. doi: 10.1371/journal.pone.0284414 (PMC10101516; doi:10.1371/journal.pone.0284414)
Supplement: S1 File — (DOCX) [file pone.0284414.s001.docx]

**S1 The details of four scales and Reliability and Validity analysis of four scales.**

**Supplement Table I** The scores of GAD-7, PHQ-9, PHQ-15 and PSQI in patients with scores≥1 (x±s)

| Scale name | Number of patients with score≥ 1 | Scores  (x±s) |
| --- | --- | --- |
| **GAD-7** |  |  |
| Feel uneasy, worried and irritable | 892 | 0.56±0.52 |
| Unable to stop or control worry | 599 | 0.41±0.60 |
| Worry too much about all kinds of things | 900 | 0.60±0.62 |
| It's hard to relax | 584 | 0.41±0.60 |
| Unable to sit quietly because of uneasiness | 267 | 0.17±0.40 |
| Become irritable or irritable | 910 | 0.61±0.61**^*^** |
| Feel as if something terrible will happen and be afraid | 352 | 0.22±0.44 |
| **PHQ-9** |  |  |
| I have no interest in doing anything. I have no interest in doing anything | 965 | 0.60±0.55**^*^** |
| Feel depressed, depressed and hopeless | 825 | 0.52±0.57 |
| Difficult to fall asleep, always awake, or sleepy too much | 798 | 0.52±0.62 |
| I often feel tired and tired | 941 | 0.60±0.60**^*^** |
| Taste bad, or eat too much | 611 | 0.41±0.58 |
| I am dissatisfied with myself, feel that I am a loser, or let my family lose their face | 521 | 0.35±0.56 |
| Unable to concentrate, even when reading newspapers or watching TV, memory decreases | 430 | 0.29±0.54 |
| Move or speak slowly enough to attract people's attention, or just the opposite, sit restlessly, be irritable and move around | 324 | 0.20±0.33 |
| Have the idea that it is better to die, or how to hurt yourself | 110 | 0.07±0.27 |
| **PHQ-15** |  |  |
| Abdominal pain | 376 | 0.23±0.45 |
| Back pain | 418 | 0.18±0.47 |
| Pain in arms, legs or joints | 426 | 0.26±0.47 |
| Dysmenorrhea and other menstrual problems | 989 | **0.61±0.59^*^** |
| Headache | 454 | 0.29±0.50 |
| Chest pain | 202 | 0.12±0.34 |
| Dizziness | 443 | 0.28±0.48 |
| Occasionally faint | 81 | 0.05±0.23 |
| Feel the heart pounding or beating quickly | 388 | 0.24±0.44 |
| Can't breathe | 204 | 0.16±0.39 |
| Pain or other problems in sexual life | 263 | 0.28±0.45 |
| Constipation, intestinal discomfort, diarrhea | 729 | 0.44±0.56 |
| Nausea, exhaust or indigestion | 500 | 0.31±0.50 |
| Feel tired or listless | 725 | 0.44±0.15 |
| Sleep problems or troubles | 593 | 0.49±0.66 |
| **PSQI** |  |  |
| Subjective feeling of sleep quality | 1223 | 0.84±0.63 |
| Time to fall asleep | 1317 | 1.12±0.83^*^ |
| Sleep persistence | 967 | 0.74±0.71 |
| Sleep efficiency | 179 | 0.15±0.40 |
| Sleep disorder | 1156 | 0.62±0.54 |
| Hypnotic drug | 17 | 0.02±0.17**^*^** |
| Daytime dysfunction | 1176 | 0.93±0.80**^*^** |

^*^*P*<0.05

**Abbreviation:** PHQ-15: Patient health questionnaire-15; GAD-7: Generalized Anxiety Disorder-7; PHQ-9: Patient Health Questionaire-9; PSQI: Pittsburgh sleep quality index.

**Supplement Table II** Reliability and Constructional Validity of PHQ-15, GAD-7, PHQ-9 and PSQI

| Scale name | Reliability analysis | |  | Structural validity analysis | | | |
| --- | --- | --- | --- | --- | --- | --- | --- |
|  | Cronbach's α Coefficient | Spearman-Brown  Half-fold coefficient |  | | KMO value | Chi-square value of Barthes sphericity test | *P* |
| PHQ-15 | 0.809 | 0.700 |  | | 0.892 | 4 720.5 | <0.001 |
| GAD-7 | 0.870 | 0.852 |  | | 0.890 | 4 280.4 | <0.001 |
| PHQ-9 | 0.825 | 0.793 |  | | 0.913 | 5 150.9 | <0.001 |
| PSQI | 0.758 | 0.783 |  | | 0.862 | 6 469.5 | <0.001 |

**Abbreviation**: PHQ-15: Patient health questionnaire-15; GAD-7: Generalized Anxiety Disorder-7; PHQ-9: Patient Health Questionaire-9; PSQI: Pittsburgh sleep quality index.

**Supplement Table III** Spearman Correlation Analysis of PHQ-15, PHQ-9, GAD-7 and PSQI Scale

|  | PHQ-15 | | PHQ-9 | | | GAD-7 | | PSQI | |
| --- | --- | --- | --- | --- | --- | --- | --- | --- | --- |
|  | *r*(95%*CI*) | *P* | | *r*(95%*CI*) | *P* | *r*(95%*CI*) | *P* | *r*(95%*CI*) | *P* |
| PHQ-9 | 0.658(0.625, 0.688) | <0.001 | | 1 | - | 0.767(0.737, 0.793) | <0.001 | 0.643(0.617, 0.672) | <0.001 |
| GAD-7 | 0.608(0.569, 0.642) | <0.001 | | 0.767(0.737, 0.793) | <0.001 | 1 | - | 0.587(0.549, 0.621) | <0.001 |
| PSQI | 0.578(0.544, 0.613) | <0.001 | | 0.643(0.617, 0.672) | <0.001 | 0.587(0.549, 0.621) | <0.001 | 1 | - |
| PHQ-15 | 1 | - | | 0.658(0.625, 0.688) | <0.001 | 0.608(0.569, 0.642) | <0.001 | 0.578(0.544, 0.613) | <0.001 |

**Abbreviation**: PHQ-15: Patient health questionnaire-15; GAD-7: Generalized Anxiety Disorder-7; PHQ-9: Patient Health Questionaire-9; PSQI: Pittsburgh sleep quality index.
