# Supplementary material for: Predictors and incidence of depression and anxiety in women undergoing infertility treatment: A cross-sectional study
Source: PLoS One. 2023 Apr 13;18(4):e0284414. doi: 10.1371/journal.pone.0284414 (PMC10101516; doi:10.1371/journal.pone.0284414)
Supplement: Attachment — Submitted filename: REVIEW PLOS ONE.docx [file pone.0284414.s002.docx]

PLOS ONE

Predictors of depressive and anxiety in women undergoing infertility treatment: a crosssectional study

--Manuscript Draft--

| Manuscript Number: | PONE-D-23-04196 |
| --- | --- |
| Article Type: | Research Article |
| Full Title: | Predictors of depressive and anxiety in women undergoing infertility treatment: a crosssectional study |
| Short Title: | Predictors of anxiety and depressive |
| Corresponding Author: | Lingyan Wang  Sichuan University West China Second University Hospital Department of Nursing Chengdu, CHINA |
| Keywords: | Anxiety and depressive symptoms, infertile women, multivariate logistic regression analysis, GAD-7, PHQ-9 |
| Abstract: | The global incidence of infertility is increasing year by year, and the psychological pressure of infertile women has been widely concerned at present. The aim of this study was to investigate the incidence of anxiety and depression in infertile women in China and explore the risk factors which might lead to anxiety and depression. A total of 1712 eligible female patients with clinical diagnosis of infertility were enrolled in this study. GAD-7 and PHQ-9 were used to evaluate the patients’ psychological status. The reliability of all scales was evaluated by Cronbach's α and Spearman-Brown half coefficient, and KMO value was calculated by factor analysis to evaluate validity. Univariate and multivariate logistic regression analysis were applied for assessing independent risk factors of anxiety and depression, respectively. The incidence of anxiety and depression in infertile women were 25.2% and 31.3%, respectively. Cronbach's α coefficients and Spearman-Brown half-fold coefficients of GAD-7 and PHQ-9 were 0.870, 0.825, 0.852 and 0.793, respectively. Univariate and multivariate logistic regression analysis showed that education level (junior college degree or above, OR:1. 6, 95% CI: 1.2-2.1, P=0.003), somatic symptoms (severe somatic symptoms, OR:15.2, 95%CI: 5.6-41.3, P<0.001), sleep quality (poor sleep quality, OR:9.3, 95% CI:4.7-18.4, P<0.001) were independent risk factors for anxiety. And age>35 years old, moderate and severe somatic symptoms and poor sleep quality were independent risk factors for depression. Somatic symptoms and poor sleep quality are both the risk factors of anxiety and depression symptoms of infertile woman. And high educated (junior college degree or above) patients are more likely to be complicated with anxiety symptoms, while elderly patients (age>35) are prone to be complicated with depression symptoms. |
| Order of Authors: | Lingyan Wang |
|  | Youyin Tang, PhD |
|  | Yuyang Wang |
| Additional Information: |  |
| Question | Response |
| Financial Disclosure  Enter a financial disclosure statement that describes the sources of funding for the work included in this submission. Review the [submission guidelines](http://journals.plos.org/plosone/s/submission-guidelines#loc-financial-disclosure-statement) for detailed requirements. View published research articles from [PLOS ONE](http://journals.plos.org/plosone/) for specific examples. | The author(s) received no specific funding for this work. |

| This statement is required for submission and will appear in the published article if the submission is accepted. Please make sure it is accurate. |  |
| --- | --- |
| Unfunded studies Enter: The author(s) received no specific funding for this work. Funded studies Enter a statement with the following details: - Initials of the authors who received each award - Grant numbers awarded to each author - The full name of each funder - URL of each funder website - Did the sponsors or funders play any role in the study design, data collection and analysis, decision to publish, or preparation of the manuscript? - NO - Include this sentence at the end of your statement: The funders had no role in study design, data collection and analysis, decision to publish, or preparation of the manuscript. - YES - Specify the role(s) played. |  |
| * typeset |  |
| Competing Interests  Use the instructions below to enter a competing interest statement for this submission. On behalf of all authors, disclose any [competing interests](http://journals.plos.org/plosone/s/competing-interests) that could be perceived to bias this work—acknowledging all financial support and any other relevant financial or nonfinancial competing interests.  This statement is required for submission and will appear in the published article if the submission is accepted. Please make sure it is accurate and that any funding sources listed in your Funding Information later in the submission form are also declared in your Financial Disclosure statement.  View published research articles from [PLOS ONE](http://journals.plos.org/plosone/) for specific examples. | The authors have declared that no competing interests exist. |

| NO authors have competing interests Enter: The authors have declared that no competing interests exist. Authors with competing interests Enter competing interest details beginning with this statement: I have read the journal's policy and the authors of this manuscript have the following competing interests: [insert competing interests here] |  |
| --- | --- |
| * typeset |  |
| Ethics Statement  Enter an ethics statement for this submission. This statement is required if the study involved:   - Human participants - Human specimens or tissue - Vertebrate animals or cephalopods - Vertebrate embryos or tissues - Field research   Write "N/A" if the submission does not require an ethics statement.  General guidance is provided below. Consult the [submission guidelines](http://journals.plos.org/plosone/s/submission-guidelines#loc-guidelines-for-specific-study-types) for detailed instructions. Make sure that all information entered here is included in the Methods section of the manuscript. | This study is a cross-sectional study which have passed the examination of Medical Ethics Committee of West China Second Hospital of Sichuan University (Ethic number: No.2020167) (Shown in S2.). Written informed consents for individuals were waived due to no individual information can be identified during the study. And a verbal consent was witnessed by the researchers and patients. |

Format for specific study types

Human Subject Research (involving human

participants and/or tissue)

Give the name of the institutional review

board or ethics committee that approved the

study

•

Include the approval number and/or a

statement indicating approval of this

research

•

Indicate the form of consent obtained

(

written/oral) or the reason that consent was

not obtained (e.g. the data were analyzed

anonymously)

•

Animal Research (involving vertebrate

animals, embryos or tissues)

Provide the name of the Institutional Animal

Care and Use Committee (IACUC) or other

relevant ethics board that reviewed the

study protocol, and indicate whether they

approved this research or granted a formal

waiver of ethical approval

•

Include an approval number if one was

obtained

•

If the study involved

non-human primates

,

add

additional details

about animal welfare

and steps taken to ameliorate suffering

•

If anesthesia, euthanasia, or any kind of

animal sacrifice is part of the study, include

briefly which substances and/or methods

were applied

•

Field Research

Include the following details if this study

involves the collection of plant, animal, or

other materials from a natural setting:

Field permit number

•

Name of the institution or relevant body that

granted permission

•

Data Availability

Authors are required to make all data

underlying the findings described fully

available, without restriction, and from the

time of publication. PLOS allows rare

exceptions to address legal and ethical

concerns. See the

[PLOS Data Polic](http://journals.plos.org/plosone/s/data-availability)

[y](http://journals.plos.org/plosone/s/data-availability)

and

[FA](http://journals.plos.org/plosone/s/data-availability#loc-faqs-for-data-policy)

[Q](http://journals.plos.org/plosone/s/data-availability#loc-faqs-for-data-policy)

for detailed information.

Yes - all data are fully available without restriction

| A Data Availability Statement describing where the data can be found is required at submission. Your answers to this question constitute the Data Availability Statement and will be published in the article, if accepted. |  |
| --- | --- |
| Important: Stating ‘data available on request from the author’ is not sufficient. If your data are only available upon request, select ‘No’ for the first question and explain your exceptional situation in the text box. |  |
| Do the authors confirm that all data underlying the findings described in their manuscript are fully available without restriction? |  |
| Describe where the data may be found in full sentences. If you are copying our sample text, replace any instances of XXX with the appropriate details.   - If the data are held or will be held in a public repository, include URLs, accession numbers or DOIs. If this information will only be available after acceptance, indicate this by ticking the box below. For example: All XXX files   are available from the XXX database (accession number(s) XXX, XXX.).   - If the data are all contained within the manuscript and/or Supporting Information files, enter the following: All relevant data are within the manuscript and its Supporting Information files. - If neither of these applies but you are able to provide details of access elsewhere, with or without limitations, please do so. For example:   Data cannot be shared publicly because of [XXX]. Data are available from the XXX Institutional Data Access / Ethics Committee (contact via XXX) for researchers who meet the criteria for access to confidential data.  The data underlying the results presented in the study are available from (include the name of the third party | All relevant data are within the manuscript and its Supporting Information files. |

| and contact information or URL).  • This text is appropriate if the data are owned by a third party and authors do not have permission to share the data.  * typeset |  |
| --- | --- |
| Additional data availability information: |  |

Manuscript	[Click here to access/download;Manuscript;manuscript.docx](https://www.editorialmanager.com/pone/download.aspx?id=32661662&guid=fefc41d4-7979-4e30-ba91-6bcf7070c789&scheme=1)

1 **Predictors of depressive and anxiety in women undergoing infertility treatment:**
2 **a cross-sectional study**

# 3 Short title: Predictors of anxiety and depressive

4

## 5 Lingyan Wang^1^, Youyin Tang^2^, Yuyang Wang^1, *^

6

7

8

9

10 1. Department of Obstetrics and Gynecology Nursing, Key Laboratory of Birth Defects and Related
11 Disease of Women and Children, West China Second University Hospital, Sichuan University, No. 20 12 South Renmin Road, Chengdu 610041, P.R. China.

13 2. Department of Vascular Surgery, West China Hospital, Sichuan University, No. 17 South Renmin 14 Road, Chengdu 610041, P.R. China

15

16

17

18 ***Corresponding author:** Yuyang Wang, E-mail: 1536803465@qq.com; Department of Obstetrics and
19 Gynecology Nursing, Key Laboratory of Birth Defects and Related Disease of Women and Children,
20 West China Second University Hospital, Sichuan University, No. 20 South Renmin Road, Chengdu 21 610041, P.R. China.

22

# Abstract

The global incidence of infertility is increasing year by year, and the psychological pressure of infertile women has been widely concerned at present. The aim of this study was to investigate the incidence of anxiety and depression in infertile women in China and explore the risk factors which might lead to anxiety and depression. A total of 1712 eligible female patients with clinical diagnosis of infertility were enrolled in this study. GAD-7 and PHQ-9 were used to evaluate the patients’ psychological status. The reliability of all scales was evaluated by Cronbach's α and Spearman-Brown half coefficient, and KMO value was calculated by factor analysis to evaluate validity. Univariate and multivariate logistic regression analysis were applied for assessing independent risk factors of anxiety and depression, respectively. The incidence of anxiety and depression in infertile women were 25.2% and 31.3%, respectively. Cronbach's α coefficients and Spearman-Brown half-fold coefficients of GAD-7 and PHQ9 were 0.870, 0.825, 0.852 and 0.793, respectively. Univariate and multivariate logistic regression analysis showed that education level (junior college degree or above, OR:1. 6, 95% CI: 1.2-2.1, P=0.003), somatic symptoms (severe somatic symptoms, OR:15.2, 95%CI: 5.6-41.3, P<0.001), sleep quality (poor sleep quality, OR:9.3, 95% CI:4.7-18.4, P<0.001) were independent risk factors for anxiety. And age>35 years old, moderate and severe somatic symptoms and poor sleep quality were independent risk factors for depression. Somatic symptoms and poor sleep quality are both the risk factors of anxiety and depression symptoms of infertile woman. And high educated (junior college degree or above) patients are more likely to be complicated with anxiety symptoms, while elderly patients (age>35) are prone to be complicated with depression symptoms.

# Introduction

Infertility is a disease of reproductive system, which is defined as the failure to conceive despite 12 months of unprotected intercourse(1). Studies have revealed that environmental pollution, food safety, alcoholism, smoking, endocrine dysfunction, abnormal immune function, delayed childbearing age and other factors will contribute to an increase risk of couples suffered from infertility(2). There are nearly 200 million people diagnosed with infertility worldwide(3). The incidence of infertility is increasing year by year in China(4). Previous studies have shown that there were more than 50 million infertile patients in China in 2014, with a prevalence rate of 15%(5). However, by 2023, the prevalence rate of infertility is expected to be as high as 18.2%(4, 6, 7).

Previous studies have shown that besides being unable to conceive naturally, infertility is also related to various physical and mental diseases such as anxiety, depression, obsessive-compulsive symptoms and sleep disorder(8, 9). In the past years, infertility was considered as a shameful disease, and society and family will give individuals and couples a greater psychological burden, which greatly affects the physical and mental health of infertile patients(10). For infertile patients, first went to outpatient consultation or chose assisted reproductive technology treatment may cause anxiety(11). In Japan, about

67% of unmarried women believe that couples should have at least one child after marriage, while about 25% of married women believe that a couple is socially acceptable only after they have children, according to the study(12). In China, people attach great importance to fertility(13, 14). Even though women have relatively high family decision-making power than in the past, there still exist a tendency in society to think that "it is always women's fault to have no children”(5, 15). As a result, compared with male patients, female infertile patients suffered more psychosocial pressure and were more likely to induce psychosocial-related physical and mental diseases, such as anxiety, depression, obsessivecompulsive symptoms and sleep disorders(16).

In recent years, with the increasing incidence of infertility, more and more researchers have begun to pay attention to the psychological stress, such as anxiety and depression, of infertile women(5, 9, 17). However, many of the current studies focus on the relationship between stigma and psychological distress(4, 5, 12, 18), only few studies(13, 14) investigate the risk factors which causes infertility women suffered from psychological stress. Besides, the sample size included in these studies are not large enough, and they only investigate the influence of demographic characteristics on psychological stress of infertile women. Thus, a large sample size study with more variables focus on psychological stress of infertile female patients is helpful for clinicians to accurately predict the high-risk factors and severity of psychological stress of infertile female patients, and help guide clinical decision-making.

Based on the above reasons, we designed a cross-sectional study to investigate the incidence of anxiety and depression symptoms of infertile female patients in West China Second Hospital of Sichuan University, aiming at selecting the risk factors causing anxiety and depression symptoms and predicting the risk of anxiety and depression of infertile female patients.

# Materials and Methods

## Study design and setting

The demographic characteristics, somatic symptoms, sleep quality and psychological stress of female infertile patients in our hospital were collected by questionnaire. Psychological stress refers to anxiety and depression. Patient health questionnaire-15 (PHQ-15) was used to record physical symptoms and Pittsburgh sleep quality index (PSQI) was used to record sleep quality. Anxiety and depression were assessed with Generalized Anxiety Disorder (GAD-7) and Patient Health Questionaire-9 (PHQ-9), respectively. In addition, anxiety and depression symptoms (converted into binary variables) were taken as dependent variables, and physical symptoms, sleep quality and demographic characteristics were taken as independent variables. Univariate and multivariate logistic regression analysis was carried out to select independent risk factors and quickly identify high-risk patients, so as to facilitate early psychological intervention.

## Patient selection

During January 2020 to December 2020, female infertile patients who received assisted reproduction technology (ART) treatment at West China Second Hospital were recruited and medical records of them were reviewed, and a total of 1712 female infertile patients were included in this study.

The inclusion criteria were: i) patient should be older than 20 years old; ii) patient had received primary school education and above and able to communicate normally; iii) patient had good physical condition and had not been diagnosed with mental illness before. Exclusion criteria: i) patient had impairment of reading, comprehension or writing; ii) patient was diagnosed with consciousness disorder and cognitive disorder of orientation.

## Questionnaire design and content

Demographic characteristics include age, educational, occupation, causes of infertility. Physical symptoms (evaluated by PHQ-15) include 15 items such as abdominal pain, back pain, arm, leg or joint pain, dysmenorrhea, sexual intercourse pain, etc., and sleep quality (evaluated by PSQI) include 7 items (sleeping time, sleep efficiency, hypnotic drugs, etc.). The scores of GAD-7, PHQ-9, PHQ-15 and PSQI in patients were shown in **S1**.

### Generalized anxiety disorder-7 (GAD-7)(19)

The scale for evaluating patients' generalized anxiety symptoms and severity in the past 1 month consists of 7 scoring items, and each item is scored according to 4 grades (0, 1, 2 and 3), with a total score ranging from 0 to 21. The higher the score, the more serious the anxiety symptoms. Grading criteria: 0 to 4 for no anxiety, 5 to 9 for mild anxiety, 10 to 14 for moderate anxiety and 15 to 21 for severe anxiety.

### Patient health questionnaire-9(PHQ-9)(20)

To evaluate the psychological condition of patients in the past 2 weeks, there were 9 items in total, and each item was scored according to 4 grades (0, 1, 2 and 3), with a total score ranging from 0 to 27. The higher the score, the more severe the depressive symptoms. Grading criteria: 0 to 4 for no depression, 5 to 9 for mild depression, 10 to 14 for moderate depression and 15 to 27 for severe depression.

### Quality Control and Reliability & Validity Analysis(21)

The two researchers who participated in the quality control of this study were trained professionals (with the national third-class and above psychological counselor qualification certificate) to ensure the authenticity and validity of the questionnaire and scale. In this study, patients do not need to fill in private information such as their names and ID numbers, and all patients voluntarily participate in this study and sign informed consent forms. At the same time, researchers are not allowed to guide, mislead or fill in on their behalf.

Cronbach's α coefficient and Spearman-Brown half-fold coefficient were used to analyze the reliability of the scale. Generally, Cronbach's α coefficient> 0.7 and Spearman-Brown half-fold coefficient> 0.7 suggesting that the internal consistency of the questionnaire is good, that is, the reliability is good. Factor analysis was used to evaluate the structure validity of each scale by calculating the KMO value. KMO value> 0.6 indicates a well data structure validity. Calibration validity refers to the proximity between the scale measurement results and the standard measurement.

## Ethical Considerations

This study is a cross-sectional study which have passed the examination of Medical Ethics Committee of West China Second Hospital of Sichuan University (Ethic number: No.2020167) **(Shown in S2.)**.

Written informed consent was waived due to no individual information was identified.

## Statistical Analysis

Continuous variables are expressed by mean ± standard deviation (x± s), and t test is used for statistical analysis. Counting data is expressed as number (n), and chi-square test is applied for statistical analysis.

The reliability of all scales was evaluated by Cronbach's α and Spearman-Brown half coefficient, and KMO value was calculated by factor analysis to evaluate validity. Univariate and multivariate Logistic analysis was used to analyze the independent risk factors leading to anxiety symptoms or depression symptoms. Variables with p-value<0.1 in univariate analysis were further stepped in multivariate Logistic

regression analysis. The test level α was 0.05. P value< 0.05 was considered statistically significant. All statistical analyses were conducted with EmpowerStats software, version 2.20 and SPSS20.0 software.

# Results

## Demographic Characteristics of patients

In this study, a total of 2301 female infertility patients were given questionnaires, among which 438 patients refused to participate in the survey, and 151 patients presented incomplete questionnaires. The flow diagram of this study was showed in Figure 1 **(Fig 1**). A total of 1712 valid questionnaires were recovered, with a questionnaire recovery rate of 74.4%. The patients ranged from 20 to 49 years old, with an average age of 32.1±4.59 years old. Besides, the incidence of anxiety and depression in infertile women were 25.2% and 31.3%, respectively. Among all the patients included in this study, there was

836 patients whose age range from 30 to 35 years old with the highest composition ratio of 48.8%. While 1118 patients had an education level of junior college or above, accounting for 65.3% of all patients. The differences were statistically significant (P< 0.05) **(Table 1).**

**Table I** The baseline characteristics of infertility women in this study

| Variable | |  | Anxiety symptoms（assessed by GAD-7） | | | |
| --- | --- | --- | --- | --- | --- | --- |
|  |  | No anxiety  (n=1267) | Mild anxiety Moderate anxiety Severe anxiety  (n=383) (n=49) (n=13) | | | |
|  | **Age, year, n** |  |  |  |  |  |
|  | (20, 29] | 428 | 132 | 12 | 5 |  |
|  | (30, 35] | 601 | 199 | 30 | 6 |  |
|  | >35 | 238 | 52 | 7 | 2 |  |
|  | **Educational level, n** |  |  |  |  |  |
|  | Junior high school | 247 | 49 | 7 | 4 |  |
|  | High school | 220 | 59 | 8 | 0 |  |
|  | Junior college degrees or above | 800 | 275 | 34 | 9 |  |
|  | **Occupational status, n** |  |  |  |  |  |
|  | Employed | 603 | 208 | 27 | 6 |  |
|  | Un-employed | 664 | 175 | 22 | 7 |  |
|  | **Causes of infertility, n** |  |  |  |  |  |
|  | Primary infertility | 557 | 184 | 23 | 5 |  |
|  | Secondary infertility | 710 | 199 | 26 | 8 |  |
|  | **Sleep quality^#^, n** |  |  |  |  |  |
|  | Very well | 819 | 91 | 2 | 1 |  |
|  | General | 431 | 257 | 33 | 4 |  |
|  | Poor | 17 | 34 | 14 | 8 |  |
|  | Awful sleep quality | 0 | 1 | 0 | 0 |  |
|  | **Somatic Symptoms*, n** |  |  |  |  |  |
|  | No | 924 | 107 | 6 | 1 |  |
|  | Mild | 302 | 170 | 18 | 3 |  |
|  | Moderate | 36 | 92 | 18 | 5 |  |
|  | Severe | 5 | 14 | 7 | 4 |  |

**Abbreviation:** GAD-7: Generalized Anxiety Disorder 7;

\# Assessed by Pittsburgh sleep quality index (PSQI);

*Assessed by Physical Health Questionnaire-15(PHQ-15).

## Reliability, Construction Validity and Spearman Correlation Analysis of the Scale

Cronbach's α coefficients of PHQ-15, GAD-7, PHQ-9 and PSQI were 0.809, 0.870, 0.825 and 0.758 respectively. Spearman-Brown half-fold coefficients were 0.700, 0.852, 0.793 and 0.783 respectively,

and KMO values were 0.892, 0.890, 0.913 and 0.862 respectively. Cronbach's α coefficients and Spearman-Brown half-fold coefficients suggested that the reliability and construction validity of the four scales included in this study are good (P < 0.001). **(Shown in S1)**

Spearman correlation analysis showed that the correlation coefficients was well (P < 0.001). (**Shown in S1)**

## Univariate and multivariate Logistic regression analysis of anxiety and depressive symptoms

Univariate and multivariate logistic analysis showed that education level (junior college degree or above, OR:1. 6, 95%CI: 1.2-2.1, P=0.003), somatic symptoms (severe somatic symptoms, OR:15.2, 95%CI: 5.6-41.3, P<0.001), sleep quality (poor sleep quality, OR:9.3, 95%CI:4.7-18.4, P<0.001) were independent risk factors for anxiety. The univariate analysis and multivariate logistic analysis of anxiety was showed in **Table 2**.

**Table II** Univariate analysis and multivariate logistic regression analysis of the anxiety in infertile women.

| Variable | | | Univariate analysis  (*p* value) | | | Multivariate analysis  OR (95%CI) | | | *p* value | |
| --- | --- | --- | --- | --- | --- | --- | --- | --- | --- | --- |
| **Age, year**  (20, 29] | | | ref | | | ref | | | ref | |
| (30, 35] | | | 0.28 | | | 1.1（0.8, 1.4） | | | 0.52 | |
| >35  **Occupational status** | | | 0.06 | | | 0.8（0.5, 1.2） | | | 0.22 | |
|  | Employed |  | | ref |  | | ref |  | ref |  |
|  | Un-employed |  | | 0.71 |  | | 1.2（0.8, 1.8） |  | 0.77 |  |
|  | **Educational background** |  | |  |  | |  |  |  |  |
|  | Junior high school |  | | ref |  | | ref |  | ref |  |
|  | Higher school |  | | 0.27 |  | | 1.4（0.9, 2.2） |  | 0.13 |  |
|  | Junior college degrees or above |  | | <0.001 |  | | 1.6（1.2, 2.1） |  | 0.003 |  |
|  | **Somatic Symptoms*** |  | |  |  | |  |  |  |  |
|  | No |  | | ref |  | | ref |  | ref |  |
|  | Mild |  | | <0.001 |  | | 3.4（2.6, 4.5） |  | <0.001 |  |
|  | Moderate |  | | <0.001 |  | | 13.3（9.3, 19.1） |  | <0.001 |  |
|  | Severe |  | | <0.001 |  | | 15.2（5.6, 41.3） |  | <0.001 |  |
|  | **Causes of infertility** |  | |  |  | |  |  |  |  |
|  | Primary infertility |  | | ref |  | | ref |  | ref |  |
|  | Secondary infertility |  | | 0.88 |  | | 1.0（0.8, 1.3） |  | 0.26 |  |
|  | **Sleep quality^#^, n** |  | |  |  | |  |  |  |  |
|  | Very well |  | | ref |  | | ref |  | ref |  |
|  | General |  | | <0.001 |  | | 3.3（2.6, 4.2） |  | <0.001 |  |
|  | Poor |  | | <0.001 |  | | 9.3（4.7, 18.4） |  | <0.001 |  |
|  | Awful sleep quality |  | | 0.97 |  | | - |  | - |  |

**Abbreviation**: ref: reference; OR: odds ratio; CI: confidence interval; *Assessed by Physical Health Questionnaire-15(PHQ-15).

\# Assessed by Pittsburgh sleep quality index (PSQI);

Univariate and multivariate Logistic analysis showed that age>35 years old, moderate and severe somatic symptoms and poor sleep quality were independent risk factors for depression. Severe somatic symptoms and poor sleep quality had a very strong contribution to anxiety, with an Odds Ratio of 13.3 and 16.1 respectively. The univariate analysis and multivariate Logistic analysis of depress was showed in **Table**

**3.**

**Table III** Univariate analysis and multivariate logistic regression analysis of the depression in infertile women.

Univariate analysis	Multivariate analysis

Variable	*p* value

(*p* value)	OR (95%CI)

| **Age, year** | |  | | |  | | |  | |  |
| --- | --- | --- | --- | --- | --- | --- | --- | --- | --- | --- |
| (20, 29] | | Ref | | | ref | | | Ref | |  |
| (30, 35] | | 0.670 | | | 1.0（0.8, 1.3） | | | 0.99 | |  |
| >35 | | 0.012 | | | 0.7（0.5, 0.98） | | | 0.044 | |  |
| **Occupational status** | |  | | |  | | |  | |  |
| Employed | | Ref | | | ref | | | ref | |  |
| Un-employed |  | | 0.167 |  | | 0.9（0.7, 1.3） |  | | 0.90 | |
| **Educational background** |  | |  |  | |  |  | |  | |
| Junior high school |  | | Ref |  | | ref |  | | ref | |
| Higher school |  | | 0.769 |  | | 1.0（0.6, 1.5） |  | | 0.88 | |
| Junior college degrees or above |  | | 0.016 |  | | 1.3（1.0, 1.8） |  | | 0.089 | |
| **Physical Symptoms*** |  | |  |  | |  |  | |  | |
| No |  | | ref |  | | ref |  | | ref | |
| Mild |  | | <0.001 |  | | 4.0（3.1, 5.2） |  | | <0.001 | |
| Moderate |  | | <0.001 |  | | 14.8（8.9, 24.6） |  | | <0.001 | |
| Severe |  | | <0.001 |  | | 13.3（4.0, 44.2） |  | | <0.001 | |
| **Causes of infertility** |  | |  |  | |  |  | |  | |
| Primary infertility |  | | Ref |  | | ref |  | | ref | |
| Secondary infertility |  | | 0.736 |  | | 0.7（0.5, 0.9） |  | | 0.99 | |
| **Sleep quality^#^, n** |  | |  |  | |  |  | |  | |
| Very well |  | | Ref |  | | ref |  | | ref | |
| General |  | | <0.001 |  | | 3.1（2.6, 3.7） |  | | <0.001 | |
| Poor |  | | <0.001 |  | | 16.1（7.1, 36.5） |  | | <0.001 | |
| Awful sleep quality |  | | 0.965 |  | | - |  | | - | |

**Abbreviation**: ref: reference; OR: odds ratio; CI: confidence interval; *Assessed by Physical Health Questionnaire-15(PHQ-15).

\# Assessed by Pittsburgh sleep quality index (PSQI);

# Discussion

We conducted a cross-sectional study to investigate the prevalence of anxiety and depression among women treated for infertility and the independent risk factors for anxiety and depression. This study found that anxiety symptoms and depression in infertile women were significantly correlated with their educational level, physical symptoms and sleep quality, rather than occupation status and infertility reasons. In addition, age> 35 years old is an independent risk factor for depression. Therefore, psychoeducation is necessary for infertile women who combined the above risk factors to reduce the risk of anxiety or depression. The main findings and impacts are discussed as follows.

Multivariate logistic regression analysis found that moderate and severe somatic symptoms were independent risk factors of anxiety (P<0.001) and depression in infertile patients (P< 0.001), and severe somatic symptoms can increase the risk of anxiety symptoms by 14.2-fold (OR:15. 2, 95% CI: 5.6- 41.3, P<0.001). Further analysis of the sources of patients' physical symptoms shows that dysmenorrhea and other menstrual problems account for 58% (P<0.05). In addition, many studies have shown that all kinds of pain can increase the risk of anxiety and depression, which were consistent with the conclusion of our study(22-24). At the same time, in a study of female dysmenorrhea, it was found that women with dysmenorrhea is also associated with anxiety and depression(25). Therefore, clinical attention should be paid to patients' dysmenorrhea and other somatic symptoms in order to reduce their risk of anxiety and depression.

Poor sleep quality can increase the risk of infertile women suffered from depression symptoms by 15.1-fold (OR:16. 1, 95% CI:7.1-36.5, P<0.001). Previous investigations(26-28) implied that insomnia can increase the risk of depression or anxiety through impair emotional regulation, which supported our result. Further analysis of PSQI scale found an interesting phenomenon: 71.8% of patients had subjective sleep disorder, but only 0.99% of patients used hypnotic drugs to help them sleep. Although we don't know why only a few patients use hypnotics to sleep, we think that the sleep quality of patients can be improved by strengthening the preaching of safe use of hypnotics to help patients sleep and guiding ~~patients~~ them to actively go to sleep medical centers.

In China, people attach great importance to fertility, and there is a tendency in society to think that

"it is always a woman's fault to have no children”(5). This study found that the incidence of anxiety and depression in infertile women were 25.2% and 31.3%, respectively, which is lower than the incidence of anxiety and depressed reported in Japan of 51.1% and 54%, respectively(12). In addition, this study found that junior college degree or above only increased the risk of anxiety symptoms (OR:1. 6, 95% CI:

1.2-2.1, P=0.003), but did not increase the risk of depression symptoms (OR:1. 3, 95% CI: 1.0-1.8, P=0.089). Although the impact of education level on anxiety is controversial, the majorities believed that higher education level will increase the incidence of anxiety disorder, especially for women, this is because the current labor market cannot provide adequate economic or social resources for people with higher education level, and this mismatch between labor and salary will lead to depression and disappointment of people with higher education, which might further result in mental illness(29). In China, with the development of economy, women usually receive good education, however, the ensuing saturation of labor market will also increase the life and work pressure of higher education people, and then bring psychological stress. Previous studies have suggested that the influence of education level on mental health is not simply increasing linearly, but there is a threshold. Once the education level is higher than this threshold, the risk of mental illness will greatly increase(30, 31), which is also consistent with the current conclusion of our research that highly educated infertile patients are more likely to have anxiety symptoms. Although we didn’t deeply explore why high education level has such a different impact on anxiety and depression, it is completely consistent with the relationship between Japanese infertile women's education level and anxiety or depression reported in Yokota's research(12), which further proves the reliability of our research conclusions. Therefore, we should provide the psychological test and offer psychoeducation for highly educated infertile women to reduce the impact of anxiety and depression on them.

Another ~~founding~~ finding was that age> 35 years old was an independent risk factor for depressive symptoms in infertile women (P< 0.05). These finding was similar to many previous study conducted in other provinces of China(13, 14). And the possible reason might be that with the increase of age, the probability of successful conception of infertile patients will gradually decrease(32). A large clinical study involving 1612 women with a total of 4246 insemination cycles showed that when the age of infertile patients is over 35 years old, the clinical pregnancy rate after intrauterine insemination will decrease from 27.1% to 21.6%(33), which may be the potential cause of depression in elderly patients. Therefore, for infertile women ~~who was~~ over 35 years old, psychological test and psychoeducation ~~were is~~ will be helpful to reduce the risk of anxiety and depression.

However, there are still some limitations in this study. Firstly, this study is a single-center cross-sectional study, and there is inevitable selectivity bias. The results of this study still need to be confirmed by multicenter prospective studies. Secondly, due to the high mobility of infertility outpatients, it is hardly to evaluate all patients again, so it will have a certain impact on the repeatability of the study. Finally, during the study period, COVID-19 pandemic broke out, and the psychological state of infertile women patients may be affected by COVID-19 pandemic, so it might have an impact on the research conclusion. However, most of the patients included in the study were from Sichuan Province where ~~which~~ the epidemic ~~is~~ was not serious ~~in this area~~. Thus, the selection bias of this study in COVID-19 pandemic was relatively low. And we will carry out a large-scale multicenter related research in order to draw more scientific conclusions and help clinicians make more comprehensive clinical decisions in the future.

# Conclusions

Moderate and severe somatic symptoms and poor sleep quality can significantly increase the risk of anxiety symptoms and depression symptoms of infertile woman. And high educated (junior college degree or above) patients are more likely to be complicated with anxiety symptoms, while elderly patients (age>35) are more likely to be complicated with depression symptoms. Therefore, psychoeducation is necessary for infertile women who combined the above risk factors to reduce the risk of anxiety or depression.

## Acknowledgments

Thanks to the research platform provided by West China Second Hospital of Sichuan University and the statistical assistance provided by West China Big Data Center.

**Supporting information** S1. S2.

## Author Contributions

Data curation: Lingyan Wang, Youyin Tang.

Formal analysis: Youyin Tang.

Funding acquisition: NA.

Methodology:Lingyan Wang, Youyin Tang, Yuyang Wang.

Writing – original draft: Lingyan Wang, Youyin Tang.

Writing – review & editing: Yuyang Wang.

# Reference

1. Chambers GM, Dyer S, Zegers-Hochschild F, de Mouzon J, Ishihara O, Banker M, et al. International Committee for Monitoring Assisted Reproductive Technologies world report: assisted reproductive technology, 2014dagger. Human Reproduction (Oxford, England). 2021;36(11):2921-34.
2. Fabamwo AO, Akinola OI. The understanding and acceptability of assisted reproductive

technology (ART) among infertile women in urban Lagos, Nigeria. Journal of obstetrics and gynaecology : the journal of the Institute of Obstetrics and Gynaecology. 2013;33(1):71-4.

3. Inhorn MC, Patrizio P. Infertility around the globe: new thinking on gender, reproductive

technologies and global movements in the 21st century. Human reproduction update. 2015;21(4):411-26.

4. Zhao Q, Huangfu C, Li J, Liu H, Tang N. Psychological Resilience as the Mediating Factor

Between Stigma and Social Avoidance and Distress of Infertility Patients in China: A Structural Equation Modeling Analysis. Psychology research and behavior management. 2022;15:391403.

5. Zhang F, Lv Y, Wang Y, Cheng X, Yan Y, Zhang Y, et al. The social stigma of infertile

women in Zhejiang Province, China: a questionnaire-based study. BMC women's health. 2021;21(1):97.

6. de Ziegler D, Gambone JC, Meldrum DR, Chapron C. Risk and safety management in

infertility and assisted reproductive technology (ART): from the doctor's office to the ART

procedure. Psychology research and behavior management. 2013;100(6):1509-17.

7. Zhou Z, Zheng D, Wu H, Li R, Xu S, Kang Y, et al. Epidemiology of infertility in China: a population-based study. BJOG : an international journal of obstetrics and gynaecology. 2018;125(4):432-41.
8. Simionescu G, Doroftei B, Maftei R, Obreja BE, Anton E, Grab D, et al. The complex

relationship between infertility and psychological distress (Review). Experimental and Therapeutic Medicine. 2021;21(4).

9. Teklemicheal AG, Kassa EM, Weldetensaye EK. Prevalence and correlates of infertility

related psychological stress in women with infertility: a cross-sectional hospital based survey.

BMC Psychology. 2022;10.

10. Zhu H, Shi L, Wang R, Cui L, Wang J, Tang M, et al. Global Research Trends on Infertility

and Psychology From the Past Two Decades: A Bibliometric and Visualized Study. Frontiers in Endocrinology. 2022;13.

11. Holter H, Anderheim L, Bergh C, Möller A. First IVF treatment--short-term impact on

psychological well-being and the marital relationship. Human Reproduction (Oxford, England). 2006;21(12):3295-302.

12. Yokota R, Okuhara T. Association between Stigma and Anxiety, Depression, and

Psychological Distress among Japanese Women Undergoing Infertility Treatment. Healthcare (Basel, Switzerland). 2022;10(7).

13. Liang S, Chen Y, Wang Q, Chen H, Cui C, Xu X, et al. Prevalence and associated factors

of infertility among 20–49 year old women in Henan Province, China. Reproductive Health. 2021;18:254.

14. Zhu C, Yan L, He C, Wang Y, Wu J, Chen L, et al. Incidence and risk factors of infertility

among couples who desire a first and second child in Shanghai, China: a facility-based

prospective cohort study. Reproductive Health. 2022;19:155.

15. Péloquin K, Brassard A, Arpin V, Sabourin S, Wright J. Whose fault is it? Blame predicting

psychological adjustment and couple satisfaction in couples seeking fertility treatment. Journal

of psychosomatic obstetrics and gynaecology. 2018;39(1):64-72.

16. Madero S, Gameiro S, García D, Cirera D, Vassena R, Rodríguez A. Quality of life, anxiety

and depression of German, Italian and French couples undergoing cross-border oocyte

donation in Spain. Human Reproduction (Oxford, England). 2017;32(9):1862-70.

17. Kamboj N, Saraswathy KN, Prasad S, Babu N, Puri M, Sharma A, et al. Women infertility

and common mental disorders: A cross-sectional study from North India. Plos one. 2023;18(1). 18. Xie Y, Ren Y, Niu C, Zheng Y, Yu P, Li L. The impact of stigma on mental health and

quality of life of infertile women: A systematic review. Plos one. 2022;13.

19. Tomitaka S, Furukawa TA. The GAD-7 and the PHQ-8 exhibit the same mathematical

pattern of item responses in the general population: analysis of data from the National Health Interview Survey. BMC Psychology. 2021;9(1):149.

20. Pranckeviciene A, Saudargiene A, Gecaite-Stonciene J, Liaugaudaite V, GriskovaBulanova I, Simkute D, et al. Validation of the patient health questionnaire-9 and the

generalized anxiety disorder-7 in Lithuanian student sample. Plos one. 2022;17(1):e0263027.

21. Alkhadim GS. Cronbach's Alpha and Semantic Overlap Between Items: A Proposed Correction and Tests of Significance. Frontiers in Psychology. 2022;13:815490.
22. Karlson CW, Alberts NM, Liu W, Brinkman TM, Annett RD, Mulrooney DA, et al. Longitudinal pain and pain interference in long-term survivors of childhood cancer: A report

from the Childhood Cancer Survivor Study. Cancer. 2020;126(12):2915-23.

23. Zhu C, Xu J, Lin Y, Ju P, Duan D, Luo Y, et al. Loss of Microglia and Impaired BrainNeurotrophic Factor Signaling Pathway in a Comorbid Model of Chronic Pain and Depression.

Frontiers in psychiatry. 2018;9:442.

24. Wu M, Chen Y, Shen Z, Zhu Y, Xiao S, Zhu X, et al. Electroacupuncture Alleviates AnxietyLike Behaviors Induced by Chronic Neuropathic Pain via Regulating Different Dopamine Receptors of the Basolateral Amygdala. Molecular neurobiology. 2022;59(9):5299-311.
25. Faramarzi M, Salmalian H. Association of psychologic and nonpsychologic factors with primary dysmenorrhea. Iranian Red Crescent medical journal. 2014;16(8):e16307.
26. Suo X, Zhang Y, Liu Q, Zhao G, Zhu Y, Liu Y, et al. A mental health survey among young

front-line clinicians in high-risk areas during the COVID-19 sporadic epidemic in China. Frontiers in psychiatry. 2022;13:872331.

27. Jaussent I, Bouyer J, Ancelin ML, Akbaraly T, Pérès K, Ritchie K, et al. Insomnia and

daytime sleepiness are risk factors for depressive symptoms in the elderly. Sleep. 2011;34(8):1103-10.

28. Hertenstein E, Feige B, Gmeiner T, Kienzler C, Spiegelhalder K, Johann A, et al. Insomnia

as a predictor of mental disorders: A systematic review and meta-analysis. Sleep medicine

reviews. 2019;43:96-105.

29. Carpinelli Mazzi M, Iavarone A, Musella C, De Luca M, de Vita D, Branciforte S, et al. Time

of isolation, education and gender influence the psychological outcome during COVID-19

lockdown in caregivers of patients with dementia. European Geriatric Medicine. 2020;11(6):1095-8.

30. Link BG, Phelan JC, Miech R, Westin EL. The resources that matter: fundamental social

causes of health disparities and the challenge of intelligence. Journal of health and social

behavior. 2008;49(1):72-91.

31. Bracke P, Pattyn E, von dem Knesebeck O. Overeducation and depressive symptoms: diminishing mental health returns to education. Sociology of health & illness. 2013;35(8):1242-

59.

32. Dinelli L, Courbière B, Achard V, Jouve E, Deveze C, Gnisci A, et al. Prognosis factors of

pregnancy after intrauterine insemination with the husband's sperm: conclusions of an analysis of 2,019 cycles. Fertility and sterility. 2014;101(4):994-1000.

33. Schorsch M, Gomez R, Hahn T, Hoelscher-Obermaier J, Seufert R, Skala C. Success

Rate of Inseminations Dependent on Maternal Age? An Analysis of 4246 Insemination Cycles.

Geburtshilfe und Frauenheilkunde. 2013;73(8):808-11.

## Figure legend

Fig 1. The flow diagram of this study.

Abbreviation: GAD-7: Generalized Anxiety Disorder 7; PHQ-15: Patient health questionnaire-15; PSQI:

Pittsburgh sleep quality index; PHQ-9: Patient Health Questionaire-9.

Fig 1	[Click here to access/download;Figure;Figure 1.tif](https://www.editorialmanager.com/pone/download.aspx?id=32661672&guid=9da86ef0-b9f9-400f-aac3-fe910537006a&scheme=1)

S1

[Click here to access/downloa](https://www.editorialmanager.com/pone/download.aspx?id=32651603&guid=1ec88e75-9f2d-4392-a0fc-c2e9cbda9fc5&scheme=1)

[d](https://www.editorialmanager.com/pone/download.aspx?id=32651603&guid=1ec88e75-9f2d-4392-a0fc-c2e9cbda9fc5&scheme=1)

[Supporting Informatio](https://www.editorialmanager.com/pone/download.aspx?id=32651603&guid=1ec88e75-9f2d-4392-a0fc-c2e9cbda9fc5&scheme=1)

[n](https://www.editorialmanager.com/pone/download.aspx?id=32651603&guid=1ec88e75-9f2d-4392-a0fc-c2e9cbda9fc5&scheme=1)

[S1.doc](https://www.editorialmanager.com/pone/download.aspx?id=32651603&guid=1ec88e75-9f2d-4392-a0fc-c2e9cbda9fc5&scheme=1)

[x](https://www.editorialmanager.com/pone/download.aspx?id=32651603&guid=1ec88e75-9f2d-4392-a0fc-c2e9cbda9fc5&scheme=1)

Supporting Information

[Click here to access/download Supporting Information](https://www.editorialmanager.com/pone/download.aspx?id=32661916&guid=caacf1ad-c966-492a-a3f7-4a00bf74f176&scheme=1)

[S2.Ethics Review Form.pdf](https://www.editorialmanager.com/pone/download.aspx?id=32661916&guid=caacf1ad-c966-492a-a3f7-4a00bf74f176&scheme=1)
