# Supplementary material for: Predictors and incidence of depression and anxiety in women undergoing infertility treatment: A cross-sectional study
Source: PLoS One. 2023 Apr 13;18(4):e0284414. doi: 10.1371/journal.pone.0284414 (PMC10101516; doi:10.1371/journal.pone.0284414)
Supplement: Attachment — Submitted filename: Response to Reviewers.docx [file pone.0284414.s003.docx]

Dear editor,

Hope you have a nice weekend. We are very glad to receive your email about our manuscript titled “Predictors of depressive and anxiety in women undergoing infertility treatment: a cross-sectional study”. And we are very grateful to the reviewers for their review of our manuscript. The following are our responses for the questions and suggestions they raised.

Best wishes,

Lingyan Wang

**Reviewer #1:**

**(i) TITLE: Depressive should be substituted with depression and this should also be done in the short title**

Thanks for your suggestion and we appreciated for your carefully review. We have changed this word.

**(ii) ABSTRACT: Lines 24-25 is not clear and should be recasted. In lines 28-29, CAD, PHQ, KMO should all be written in full 1st. The authors should include a summary of how the patients were selected and state the study design here. Lines 39-42, it is not clear if the authors are reporting results and there is not conclusion in the abstract.**

Thanks for your suggestions and we appreciated for your carefully review. We have recasted the abstract. And since “the format criteria of PLOs One” the conclusion can be found in the last two paragraph of abstract “Somatic symptoms and poor sleep quality are both the risk factors of anxiety and depression symptoms of infertile woman. And high educated (junior college degree or above) patients are more likely to be complicated with anxiety symptoms, while elderly patients (age>35) are prone to be complicated with depression symptoms.”

**(iii) INTRODUCTION: There are several grammatical errors and wrong use of tenses that make several statement difficult to understand. The statements in the following lines are not clear and should be recasted, 56-58, 58-59, 64-67, 70-72, 74-76, . Line 78 should be --- determining the risk factors---.**

Thanks for your questions and suggestions and we appreciated for your carefully review. We have revised our manuscript.

**(iv) METHODS: Several grammatical errors and wrong use of tenses. How were the patients selected and how did the authors determine their sample size? The questionnaires should all be referenced. Line 112 should read --- severity, one month prior to the study----. Who distributed the questionnaires, how were they administered, when and where was this done and what language were they? Under ethical considerations, it is not clear if approval for conduct of the study was given by the ethical review committee and in line 138, what individual information are the authors referring to?**

Thanks for your questions and suggestions and we appreciated for your carefully review. We have revised our manuscript.

For patient selection, all patients who came to our hospital for infertility counseling during January 2020 to December 2020 were asked to finish the questionnaires and the inclusion and exclusion criteria were shown in the manuscript. EmpowerStats software was used to calculate the sample size of the study. Power analysis of the study were shown in the following table. We chose the lowest hazards ratio (college degrees or above:1.6) to calculate the estimated samples size, and the estimated samples size should be more than 914.

| **Anxiety/No anxiety** | value | **Depression/ No depression** | value |
| --- | --- | --- | --- |
| Two-sided α | 0.05 | Two-sided α | 0.05 |
| The incidence of anxiety in infertile women | 0.252 | The incidence of depression in infertile women | 0.056 |
| Assuming hazard ratio of Junior college degrees or above | 1.6 | Assuming hazard ratio of general sleep quality | 3.1 |
| Power | 90% | Power | 90% |
| Estimated patients number in this study | 914 | Estimated patients number in this study | 136 |

The questionnaires were in Chinese format and distributed by a nurse who have obtained a certification of National Psychological Consultant, her name was Lingyan Wang. And all the questionnaires were finished in the infertility counseling department of West China Second Hospital. All the questionnaires were locked down in a special box, and counted every month by Lingyan Wang, Youyin Tang and Yuyang Wang.

Written informed consent was waived due to no individual information (such as patient’s name, ID card number, phone number and biometrics identification data) was identified.

**(v) RESULTS: It is not clear in lines 159-160 which differences were statistically significant.**

Thanks for your question and we appreciated for your carefully review. In this sentence, we mean to say that the age and education level had significant difference in no anxiety patients. And we add a “*” in table 1 to express this statistically difference.

**(vi) DISCUSSION: There is no need to repeat p values or odds ratios that are in the results here. There are also very many grammatical errors and wrong use of tenses that make many sentences difficult to understand. The following sentences should be recasted for clarity; lines 212-213, 216-217, 219-220, 227-229, in 247 what do the authors mean by -- another founding--?. Lines 254-255 and 258-259 are not clear.**

Thanks for your question and we appreciated for your carefully review. We have revised our manuscript according to the above questions.

**(vii) REFERNCES. The authors should crosscheck the journal title of reference number 2. The following references do not seem complete; 8,9,10,12,17 and 18**

Thanks for your question and we appreciated for your carefully review. We have checked our references and revised some of them.

**Reviewer #2:**

**(i) I suggest to include the incidence in the title because incidence was mentioned as one of the objectives.**
Thanks for your suggestion and we appreciated for your carefully review. We have changed the title.

**(ii) Discussion
the rate (incidence of the anxiety was not discussed. Authors jumped directly to associated factors. Incidence have to be compared with previous studies and to discuss who there is difference between the other studies.**

Thanks for your suggestion and we appreciated for your carefully review. Actually, we have discussed the incidence of anxiety and depression and compared with previous studies in Line 225-227 in Discussion part. Besides, we added more details of the incidence and adjusted the position of this paragraph.

**(iii) I think sleep problem or pattern are not predictors for anxiety and depression but are effects. e,g are manifestation for anxiety and depression.**

Thanks for your question and we appreciated for your carefully review. We agree with your opinion that sleep problem might be manifestation for anxiety and depression. In this study we only want to explain the relationship between sleep problem and anxiety or depression, and we noticed that poor sleep quality patients had a strong association with depression. Therefore, we revised our manuscript according your suggestion.

**Reviewer #3:**

**(i) The article is an interesting one. The methodology is not detailed enough. For instance, the administration of the questionnaire was not clearly described. I got to know about it in the result section.**

Thanks for your suggestions and we appreciated for your carefully review. We have revised our manuscript according your suggestion. The administration of the questionnaire were as follows.

“The questionnaires were in Chinese format. A nurse (Lingyan Wang) with a National Psychological Consultant certification distributed and retrieved the questionnaires. And all the questionnaires were finished in the infertility counseling department of West China Second Hospital during January 2020 to December 2020. All the questionnaires were locked in a special box that could only be opened by Yuyang Wang. A monthly questionnaire count was performed independently by Lingyan Wang, Youyin Tang, and Yuyang Wang.”
